# Human Factor Analysis (HFA) Based on a Complex Network and Its Application in Gas Explosion Accidents

**DOI:** 10.3390/ijerph19148400

**Published:** 2022-07-09

**Authors:** Guirong Zhang, Wei Feng, Yu Lei

**Affiliations:** 1 School of Public Administration, Central South University, Changsha 410017, China; zhangguirong@csu.edu.cn (G.Z.); 185511063@csu.edu.cn (Y.L.); 2 Center for Social Stability Risk Assessment, Central South University, Changsha 410017, China

**Keywords:** human factor analysis (HFA), complex network, occupational safety

## Abstract

Humans are at the core of the social-technical system, and their behavioral errors affect the reliability and safety of the entire system in varying degrees. Occupational accidents and large-scale industrial accidents are often attributed to human errors, accounting for more than 80% of accidents. In view of the complexity of systems and the coupling of elements, a new HFA method is proposed based on a complex network. According to system safety theory, a complex network is regarded as a network composed of humans, matters, environments, and management, and the basic structure of the HFA network is summarized. On this basis, a system safety method of HFA is developed which proposes a universal human error causation model. Moreover, a network analysis method for human errors is also presented, which is a comprehensive analysis of human errors that have occurred. Finally, the above methods are applied to gas explosion accidents that occurred in China. Results show that the two methods proposed are universal to all fields, and their combination improves the effectiveness of human error management and promotes the targeted, proactive, systematic, and dynamic prevention of critical nodes and paths from a holistic perspective.

## 1. Introduction

For the vast majority of technical systems, there are people involved at every stage of the lifecycle, from design to manufacturing, operation, management, maintenance, system upgrading, or obsolescence [1]. As a result, the development of society is inseparable from the operation of a system; the operation of the system needs people’s participation and promotion. However, studies have shown that occupational accidents and large-scale industrial accidents are often attributed to human errors [2], accounting for more than 80% of accidents [3]. According to statistics, nearly 70–80% of major accidents in high-risk industries can be attributed to human error [4]. There are also related statistics for the power industry [5], road traffic [6], and other industries, showing that human error has long been the main participant and facilitator of accidents. Therefore, it is imperative to try to find more new ways to control human errors.

**Figure 1:**
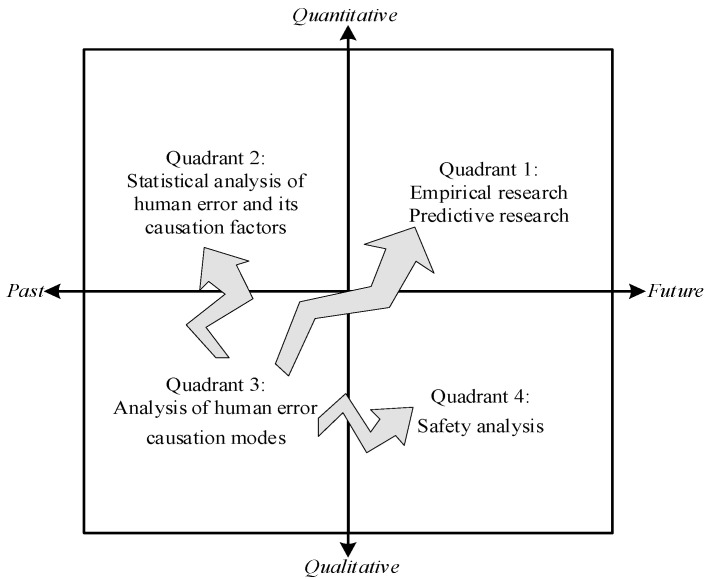
Research contents of human factor analysis.

According to past studies, HFA is a qualitative and quantitative analysis of the causation factors and paths of unsafe behaviors or human errors, thus providing a basis for safety management and the improvement of human reliability. From the two dimensions of research methods and time, relevant studies were divided into four quadrants, as shown in Figure 1, and explained as follows:

(1) Quadrant 1 is future-oriented quantitative research, mainly including safety prediction research and exploratory empirical research. This section mainly focuses on empirical research, involving empirical research on the influencing factors of human safety behavior, such as job burnout [7] and the influence of safety climate on safety behavior [8,9].
(2) Quadrant 2 is quantitative studies based on existing data, which mainly analyze the frequency, main influencing factors and paths of target human errors through the study of accident cases. Existing studies mostly focused on the analysis of the occurrence frequency of human errors and their causative factors, such as in [10,11]. A few studies conducted a more in-depth comprehensive analysis on the path of human errors, such as [12].
(3) Quadrant 3 is based on the qualitative analysis of the past, focusing on the mode of analysis of human error. The representative one is the human factor analysis and classification system (HFACS) proposed by scholars in [13], which describes the causes of human errors at four levels: ➀ organizational influence; ➁ unsafe supervision; ➂ the preconditions of unsafe behavior; and ➃ the unsafe behavior of actors. This method has been applied to civil aviation [14], maritime [15], coal mining [16], railway [17], and other systems to facilitate the investigation and analysis of accidents and its human errors. As a result, further improved or more applicable human error analysis models were generated, such as HFACS-MA [18], HFACS-OGI [19], HFACS-CM [16], etc. In addition, there is another HFA model from the perspective of information and cognition [2,20,21]. In recent years, with the development of safety information cognition, this perspective also developed from the micro-level to the macro-level. Needless to say, HFAs from different perspectives have different emphases. To improve the systematicness and pertinence of human error prevention, we should treat them with an inclusive attitude.
(4) Quadrant 4 is future-oriented qualitative analyses, which systematically analyze the possible influencing factors in the system on the basis of the existing human causation model, as to reduce the influence of the uncertainty of complex systems on safety behavior. The final results are often presented in an HFA list, e.g., as in [2,22].

Therefore, of the above four quadrants, Quadrant 3 is the premise for the others to conduct research, whereas Quadrants 1, 2, and 4 are the keys that really play a role in reality. There are two paths to carry out HFA research. One is to carry out relevant research in Quadrants 1, 2, and 4 on the basis of the existing human error causation models. The other is to propose a new, more appropriate model of human error, on the basis of which other quadrant research can be carried out.

Many complex systems in nature and society can be described in terms of networks that capture complex networks of connections between the units of which they are composed [23]. Due to the mediating effects of material, information, energy, and behavior factors, most elements of production and life are involved in complex network systems, such as the transportation system, communication system, energy system, financial system, medical system, and so on [24]. At the same time, it also includes other comprehensive systems, such as the safety system, which includes humans, facilities, equipment, environments, researchers, and managers [25]. Especially with the complexity and modernization of the system, due to the dependence of system components, risk management and safety development of complex network systems has become a huge challenge. As a result, the negative effects of cascading effects become more prominent, leading small mistakes to become big problems and local risks to evolve into large-scale disasters. Complex network analysis has been applied to many scientific fields in the past [26]. For the complex network of the safety system, complex network analysis can clarify all the factors that may affect system safety and their relationship. At the same time, it can also identify the core nodes, core associations, and risk transmission paths in the network through quantitative analysis, which previous studies have found difficult to solve [27].

**Figure 2:**
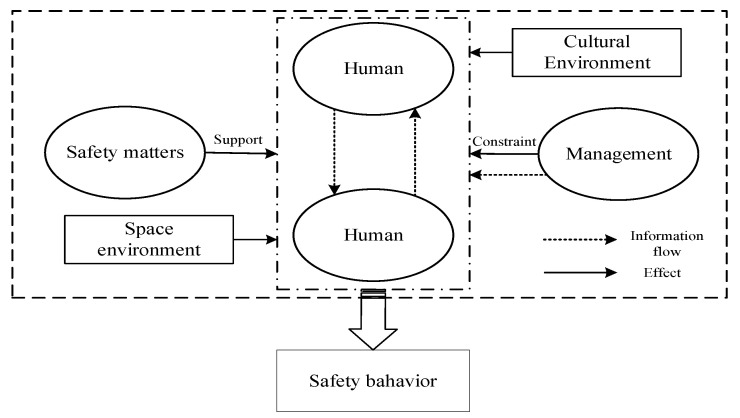
Human-centered safety system network.

In the human-centered safety system network, the influencing factors of safety behavior are extremely complex. Safety behavior is affected by its own factors, as well as by the external environment, information, and management, and also depends on related safety matters [2,20,28], as shown in Figure 2. In this complex network, the above nodes are only directly connected with people, and other nodes in the network may indirectly affect safety behavior by influencing the above nodes on the basis of complex coupling and cascading effects. In the past, relevant research of the indirect effects mentioned above were scarce and incomplete. In view of this, this research is carried out along the second path proposed above (Quadrant 3 → Quadrant 1, 2, and 4) from the perspective of a complex network system. This study first puts forward the corresponding HFA network, and then carries out the research of Quadrant 2 and Quadrant 4, and finally, carries out the application to further reduce the human error rate. In theory, a comprehensive and systematic model of human error causation is proposed; in practice, an effective methodology is provided to deal with human error in a complex system in advance.

## 2. HFA Network

The system safety theory suggests that there is nothing absolutely safe in the world, and risk factors lurk in any human activity [25]. The potential risk factor that can cause accidents was called the hazard source (or potential hazard),which includes unsafe states of matters, human mistakes, bad environmental factors, and management defects [25]. From the perspective of a complex network, the safety system can be understood as a complex network composed of humans, matters, environments, management, and their coupling effects. Each node represents a safety element in the system; the directed edge represents the influence of one node on the safety state of another node. The dependence between nodes is the premise of the network. For the purpose of HFA, the complex network can also be regarded as an HFA network. From the perspective of the relationship between subjects, HFA mainly has two influencing factors, information and constraint, as the inadequacy of communication leads to continuous unsafe behavior [2,20], and lack of superior restraint (such as supervision, management, etc.) leads to unsafe behaviors of subordinates [28]. From the perspective of humans, the occurrence of human error is actually the external manifestation of the internal cognitive process, and its influencing factor was called safety signal noise (the adverse factor in the environment that affects the correct cognition of the system safety status) [2]. Therefore, from the comprehensive perspective of the social-technical system combined with a complex network, this study divides the HFA network into three types: an HFA network based on signal-noise dependence, an HFA network based on information dependence, and an HFA network based on constraint dependence.

### 2.1. HFA Network Based on Signal-Noise Dependence

**Figure 3:**
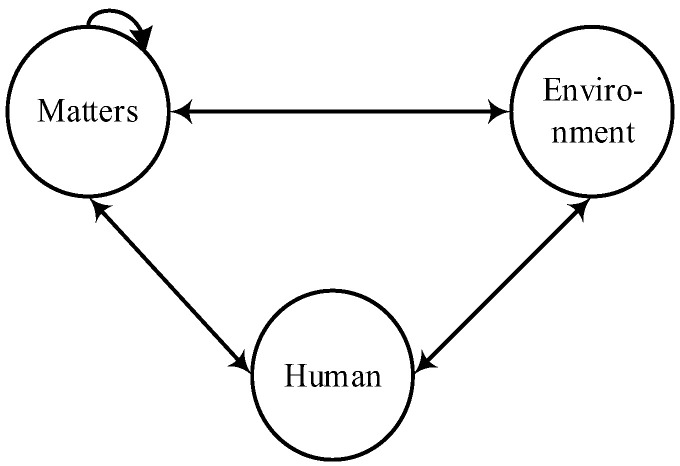
HFA network based on signal-noise dependence (solid arrows indicate the influence paths between nodes).

Signal noise is also the influential factor in the process of safety information cognition (SIC), which comes from humans, environments, and cognitive objects [2]. Among them, the SIC process was expressed as “safety information—safety perception—safety cognition—safety execution”; failure of any link results in human errors [2]. ➀ A human’s physical and psychological state, perception of environment, and the complexity and ambiguity of perception objects lead to errors, inadequacy, or inappropriateness of safety perception. ➁ Since the SICP is carried out under the support of the safety ability, the defect of the safety knowledge structure is an important factor that leads to the generation of wrong cognitive information. In addition, the psychological and physiological factors, cognitive environment, reasoning, learning, and cognitive abilities of the human are also the influencing factors [29]. ➂ The physiology and psychology, safety behavior ability, and execution environment may cause the safety execution to fail. However, the indirect factor of human errors must not be ignored in the coupling system. To further solve this complex problem, an HFA network model based on signal-noise dependence (as shown in Figure 3) is constructed and combined with the system safety theory, which is elaborated as follows:

(1) In a safety system, humans mainly include safety managers and operators, and their behaviors include the basic safety behaviors of life and production, summarized as safety interaction behaviors (human–matter interaction, human–environment interaction), which are the direct or driving factors of accidents. The state maintenance and function of environment and matters depends on safety behavior [29].
(2) Environment is the medium and state of human and matters in the system, which mainly refers to the space environment of the system, involving temperature, humidity, noise, light and shade, cleanliness, and so on. For example, high temperatures may cause device faults; too dim lighting can make it hard to find the potential hazard.
(3) Matters are not only related to production, but also to safety, such as safety facilities, safety equipment, etc., which are important supports for safety perception, cognition, and execution. Matters and the environment are mutually coupled—matters can impact, change, or control the environment of the system, and the environment then affects humans and organizations in the system. In addition, due to the correlation between physical structures, cascading effects may occur between physical structures, thus causing damage to safety matters [30].

### 2.2. HFA Network Based on Information Dependence

**Figure 4:**
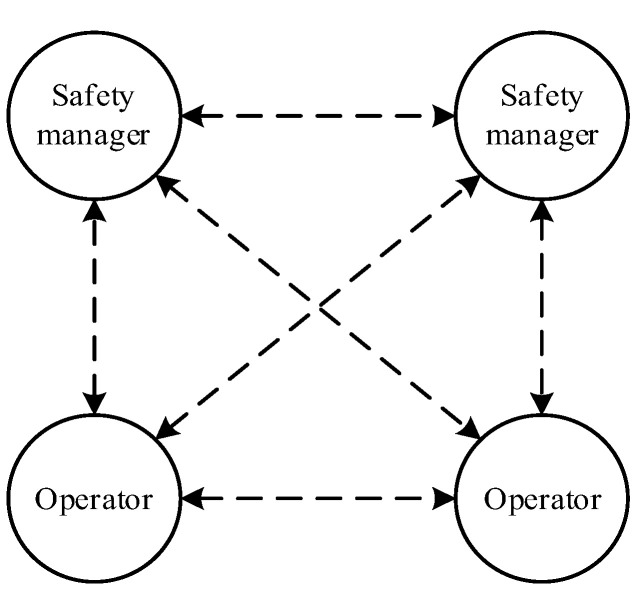
HFA network based on information dependence (dotted arrows indicate the information paths between nodes).

Information is an important factor for the generation of complex networks, and the information dependence among subjects ties them together closely. Safety information dependence mainly refers to how safety behavior of agents in the complex network depends on timely and effective safety communication. Safety communication involves a two-way exchange of information between individuals to make decisions about how best to manage risk and crisis, with the goal of avoiding the occurrence of unsafe behaviors in oneself or in others [31]. Therefore, effective safety communication within a safety system occurs vertically and horizontally, mainly involving three risk communication modes: safety information supply (SIS), safety information feedback (SIF), and safety information exchange (SIE). As a consequence, the relevant HFA network is formed, as shown in Figure 4 and described as follows:

(1) SIS. The lack of safety information caused by SIS is a common cause of human accidents [32]. It can be understood as the timely transmission of useful information by the manager to the subordinate. In the whole process of safety production, SIS from safety managers to operators is one of the most important links to prevent human errors, as the information mainly involves safety publicity, safety education, and safety training. Open and frequent SIS is significant as it helps workers to make sense of conflicting priorities, reduces ambiguity, and provides a basis for consensus about appropriate ways of working [31].
(2) SIF. SIF refers to the new regulations of the safety state of the system made up of humans and man-made subsystems in the form of safety information that adjust the safety structure, safety function, and safety behavior in the system [33]. Among them, SIF has positive incentive and reverse error correction effects (i.e., positive feedback and negative feedback) for human behavior. Especially for negative feedback, timely SIF promotes timely improvement and optimization of safety operation behavior and safety management behavior. On the contrary, untimely, inadequate, or incorrect SIF leads to the failure of the subject to find their own behavior defects.
(3) SIE. SIE is an exchange of views between subjects on a specific risk issue. This process includes three links of raising questions, transferring safety information to the target subject, and outputting relevant knowledge, opinions, and other information to the target subject. It actually covers the above two safety communication modes (i.e., the link of information output is SIS or SIF, or the combination of both). However, the difference is that this mode is led by active safety communication, whereas the first two are led by passive safety communication.

In the HFA network, SIS refers to the one-way information flow from safety manager to operator; SIF is a one-way information flow from the subject to the target subject on the basis of behavioral cognition; and SIE is the only two-way information flow, which receives the information supply and/or feedback from the target subject on the basis of the subject’s communication of active information. The above mode runs through the whole process of safety production, and an error in any link may lead to serious human accidents.

### 2.3. HFA Network Based on Constraint Dependence

Constraint means restriction and control, and it is the most fundamental and authoritative influence factor for safety behavior. In the current hierarchical structure, the restraint of the subordinate by the superior is fundamental for the normal operation of the safety governance system. Based on the constraint relationship between subjects, the safety constraint (SC) is summarized as the regulation-based SC and the management-based SC according to the operating process and governance mode of the social-technical system, where both constraints are passed down one level in the hierarchy.

(1) A safety rule is a safe state of the system or a safe manner of acting in response to a forecast, established before an event, and imposed by the operators and managers of the micro-system as a means of improving safety or achieving the required safety level [34]. As a subset of safety rules, safety regulations are rules implemented by administrative agencies or independent agencies with legal effects, which influence safety behaviors by clarifying what behaviors are illegal and the punishment of violations [35]. These rules promote individuals and organizations to avoid the occurrence of violations in the form of laws and regulations, departmental rules, industry regulations, policies, safety standards, and enterprise regulations.
(2) The essence of regulations is that they are imposed by an authority on an organization or individual that must comply and are backed by some form of institutionalized sanction for noncompliance [36]. Additionally, that management is an important part of the system in which responsibilities and codes of conduct are assigned to safety-related individuals and organizations. Therefore, the management-based SC is based on the role of the regulation-based SC and restricts safety behavior through supervision, inspection, auditing, and safety management, to specifically clarify what should be, cannot be, and how to implement SCs that involve humans, substances, machines, facilities, craft processes, environment, management, etc.

**Figure 5:**
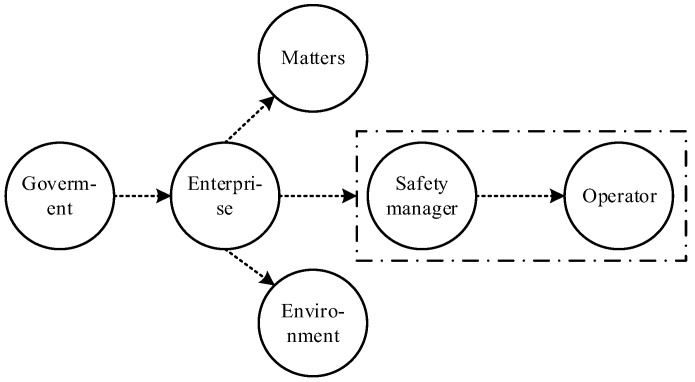
HFA network based on constraint dependence (short, dotted arrows indicate the constraint paths between nodes).

According to the functions and responsibilities of different agents, an HFA network based on constraint dependence is constructed as shown in Figure 5. Among them, the government mainly directs constraints on enterprises (including safety standards, safety regulations and safety supervision); on this basis, enterprises constrain the responsibilities of safety managers, safety matters, and safety environments in enterprises through safety investment, safety regulations, and the implementation of safety standards; finally, safety managers supervise the safety behaviors of operators in daily safety operations. Undoubtedly, the constraint failure of any link may lead to a cascading effect in the hierarchical structure, thus resulting in human error.

### 2.4. HFA Network from a Comprehensive Perspective

**Figure 6:**
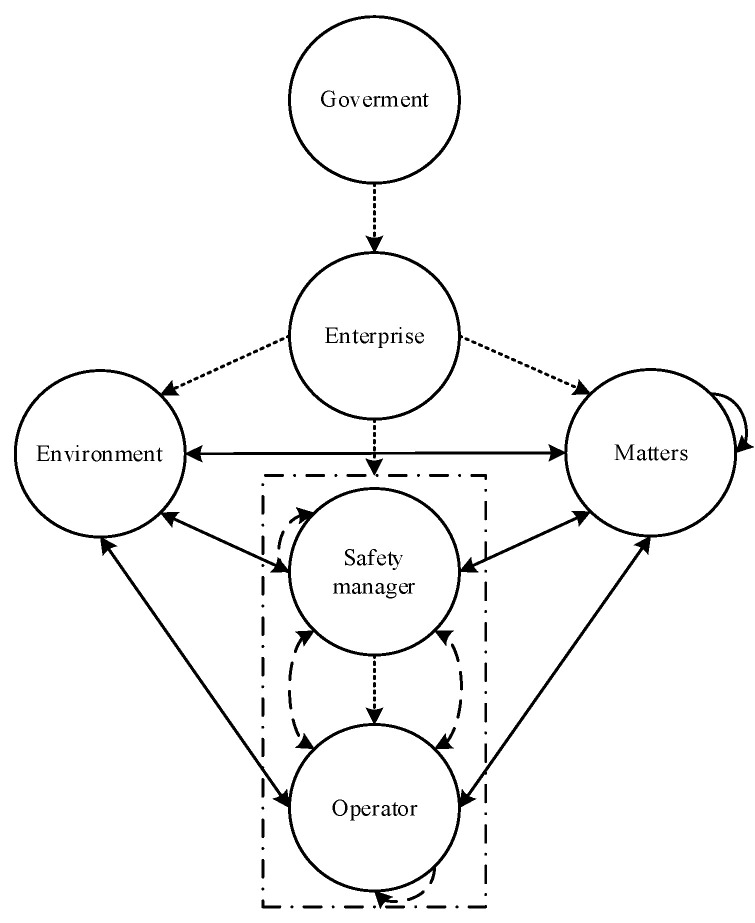
HFA network from a comprehensive perspective.

Obviously, these three kinds of HFA networks do not exist in isolation. An HFA network based on constraint dependence belongs to the HFA from the macro-perspective, which has a fundamental impact on humans, matters, and environments in the micro-system. It cannot only directly cause human error, but also reduce the timeliness and comprehensiveness of safety communication by affecting humans, or hinder individual safety perception, cognition, and execution by affecting objects and environments. Therefore, the HFA network from a comprehensive perspective is constructed as shown in Figure 6.

The result shows that the HFA network has the following distinct characteristics of a complex network: (1) Small-world network. The path between other nodes and target nodes is short, indicating the vulnerability of safety behaviors. (2) Scale-free network. Due to the complexity of the system, any target node has a dense network centered on it, which increases the vulnerability of safety behaviors. (3) Internal connection and external connection. A giant system is composed of distributed subsystems, which are not isolated but interact with each other. Therefore, HFA networks are not limited to local systems. It also highlights the vulnerability of safety behavior. To promote the comprehensive, dynamic, and fundamental development of HFA, a new method from the perspective of the complex network should be proposed to comprehensively reduce the probability of human errors based on the above four HFA networks.

## 3. System Safety Method of HFA Based on Complex Network and Its Application

The purpose of the system safety analysis method is to analyze the risk factors in the system from the perspective of safety, mainly by analyzing the various factors leading to system failure or accident and their correlation [25], and thus, leading to the formulation of countermeasures. There have been some system safety analysis methods in the past, such as the safety checklist, preliminary hazard analysis (PHA), failure mode and effect analysis (FMEA), hazard and operability study (HAZOP), event tree analysis (ETA), and fault tree analysis (FTA). These methods cover the four kinds of attributes, induction, deductive, qualitative, and quantitative, and became the important methods and tools for conducting safety research.

### 3.1. Proposal of the Method

**Figure 7:**
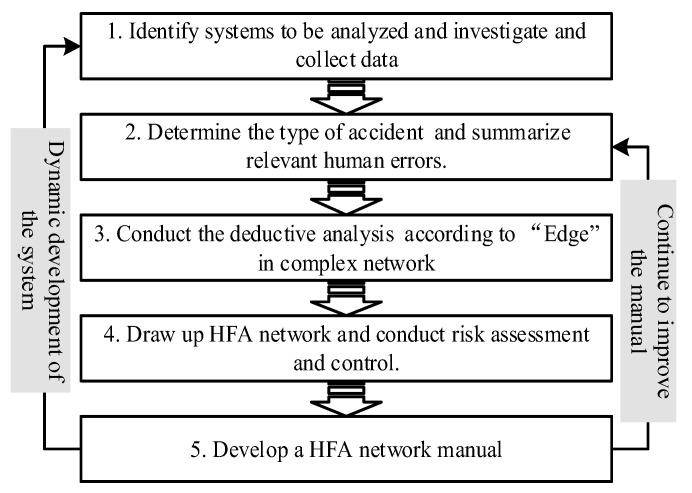
Procedure of the system safety method of HFA based on complex network.

The system safety method of HFA refers to the safety analysis of the influencing factors of human error in the operation system, which helps to put forward effective management measures. This research scenario is similar to FMEA in that it systematically analyzes the possible causes of “system failure” to formulate countermeasures. Therefore, under the guidance of the HFA network and combined with specific situations, this study intends to conduct a system safety analysis on unsafe behaviors leading to accidents (the analysis process is shown in Figure 7). The details are as follows:

(1) Identify systems to be analyzed and investigate and collect data. A system is an organic whole with specific functions combined with several components that are interdependent and interact with each other [25]. In different systems, the system structures differ significantly. Therefore, the boundary of the micro-system to be analyzed should be divided, and on this basis, all the relevant social and physical structures and their dependence relationships in the system should be clarified. Finally, the relationship between enterprises and relevant government departments is clarified from a macro-perspective.
(2) Determine the type of accident to be studied and summarize the human errors that can lead to the accident. When determining the influencing factors of a specific accident, a causal analysis should be used for inference and deduction, such as the FTA or fishbone diagram, to summarize all the human errors that may lead to the accident.
(3) Based on the “directed edge” in the complex network, deductive analysis is conducted from the perspective of specific human error, and all the causative factors and causative paths are summarized. In the HFA of target human factor errors, we plan to analyze them in stages from bottom to top based on the HFA network shown in Figure 5. There is a cascading effect among the influencing factors of the adjacent side of the department, thus forming a specific human error causation path.
(4) Draw up an HFA network and carry out risk assessment and control. Based on influencing factors and the causative path of human error, an HFA network is drawn, which facilitates the development of the current assessment of human error. Risk assessment focuses on two perspectives: on the one hand, assess the vulnerability of each node; on the other hand, evaluate the influence degree of each node in the network on human error. On this basis, take measures to control the risk.
(5) Develop an HFA network manual. To reduce the probability of human error, a targeted HFA network manual is developed according to specific subjects, where all related factors and paths that may lead to human error can be clarified, and specific countermeasures can also be put forward. To adapt to the dynamics of the system, the HFA network manual should be updated accordingly. When it is found that part of a causation factor or type of accident has not been taken into account, additional consideration shall be given.

Of course, at the macro-level, the optimization of governance behavior is the fundamental variable of human error control, which needs a targeted response by combining current governance policies and existing problems.

### 3.2. Application of the Method

In this paper, the method is applied to a coal mine gas explosion accident. The coal mining system is a complex social and technical system, involving laws and regulations, safety management, miners, equipment, geology, and other aspects [37]. In the past, gas explosions have become the main accidents that damage life and property, and risk safety in the coal mine system. The analysis of human error in gas explosion accidents has become the focus of scholars. Chen et al. analyzed and compared the characteristics of human error in gas explosions in China during 1980–1999 and 2000–2010 [37]; Yin et al. studied the characteristics of fatal gas explosion accidents and unsafe behaviors in Chinese coal mines from 2000 to 2014 from the three dimensions of accident site, operation technology, and equipment installation [11]; Tong et al. assessed the unsafe behavior of four types of miners in gas explosion accidents in China, and showed that ventilation caused the highest risk of unsafe behavior, followed by gas prevention and fire suppression [22]; Meng et al. used the FTA method to evaluate the unsafe behaviors in coal mine gas explosion accidents [38]. However, all the above studies are confined to the quantitative study of the unsafe behavior or human error itself in the gas explosion, and there is a lack of reasoning and deduction of the possible causes of human error. Our method can not only systematically summarize the human error causation factors in coal mine gas explosion accidents, but also analyze and predict the causation path. The following application focuses on step 3 and step 4:

(1) Deductive analysis of human error causes and paths: taking gas inspectors as an example

In the past, the HFACS was used to analyze the causation factors of human errors, which summarized 19 types of influencing factors of human error from the perspective of organizational influence, unsafe supervision, the preconditions of unsafe behavior, and the unsafe behavior of actors [13]; SICHFA divided the cause factors of human error into 4 categories and 10 subcategories [2]. Therefore, on the basis of the above research, this paper summarizes the causes and paths of human error from the perspective of a complex network. Since the influence paths of the operator and manager’s behaviors are different, we put forward separate causation factors and paths for each, and then carry out the specific application. The results are shown in Table 1. According to the results, there are 32 causation factors for an operator’s behavior error, and 31 causation factors for a safety manager’s behavior error.

The gas inspector is an important line of defense to prevent gas explosion accidents, which was demonstrated by the FTA of gas explosion accidents [38], and their safety responsibilities are as follows: (1) be responsible for gas drainage and monitoring in the responsible area, and check and maintain the working conditions of safety facilities for gas explosion prevention; (2) be able to timely find the hidden dangers in the operation of ventilation systems, fire protection facilities, local ventilators, the implementation of the blasting system, and the management of explosive products in the responsible area, and report the problems to the ventilation dispatcher in time; (3) when the gas exceeds the limit, stop the work at the site, find out the reason, take measures to deal with it, and report to the ventilation dispatcher. Gas inspectors are full-time safety inspectors rather than special safety management personnel; thus, they are classified as operators in this paper. Based on this, the analysis results of the causes of gas inspectors’ behavior errors are shown in Table 2. This result is a general result of the cause factors of gas inspectors applicable to any coal mine, and it should be further applied to specific people, matters, environments, and management on the basis of data collection. Needless to say, the summary of relevant causation factors provides guidance for the development of practical work.

(2) HFA network construction and risk management

**Figure 8:**
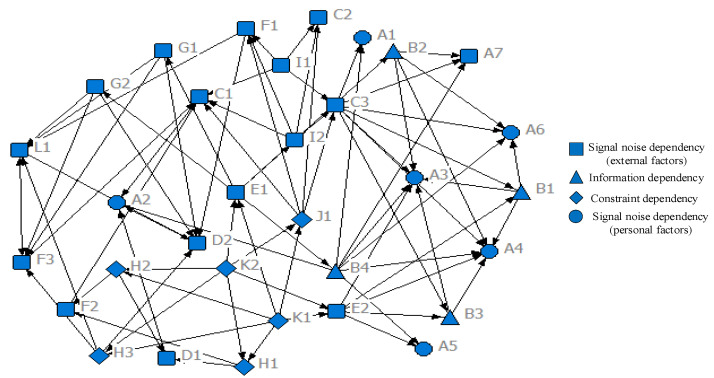
Behavior error causation network of gas inspector.

This part continues to take the gas inspector as the object and study the construction of an HFA network and its risk management based on the analysis results in Table 2. The HFA network is shown in Figure 8, which was constructed on the basis of the three dependency types in Section 2. The difference is that this part divides the signal-to-noise dependence into the signal-to-noise dependence influenced by external factors and the signal-to-noise dependence influenced by personal factors. On this basis, human error risk assessment and control are elaborated as follows:

On the one hand, the vulnerability assessment of each node is carried out by comparison with the actual situation and standards. For example, do gas inspectors have the knowledge and skills to do the job? Do gas inspectors have physical and psychological defects that hinder their work? Are underground safety facilities and equipment adequate? Is the environment in a safe state? Is the enterprise’s safety climate good? On the basis of the above evaluation, the nodes with high vulnerability should be optimized to a safe level or higher.

On the other hand, it is necessary to clarify the importance of each node in the network, that is, to clarify the key influencing factors of gas inspectors’ behavior errors. In this study, all nodes have a certain degree of influence on human error. It can be determined that, in the existing hierarchical system, the influence range of nodes based on constraint dependence, information dependence, and signal-noise dependence decreases step by step. Therefore, we further use two evaluation values that can represent the influence degree of nodes on the network, including degree centrality (involving in-degree centrality and out-degree centrality in the directed network) and betweenness centrality. Among them, in-degree centrality is used to measure the influence degree of other nodes on the target node; the out-degree centrality is used to measure the degree of influence of the target node on other nodes; the betweenness centrality measures the “bridge” effect in the HFA network [39]. The results show that A3 (6), A4 (6), C1 (5), D2 (5), and L1 (5) are the important nodes in in-degree centrality and B4 (7), C3 (7), K1 (6), K2 (6), and E2 (5) are the important nodes in out-degree centrality. The top five nodes in betweenness centrality are C3 (25.167), E1 (13.733), J1 (10.567), E2 (10.500), and F1 (9.000). Therefore, government should strengthen safety supervision or adopt policies and institutions that can promote government supervision, such as the use of incentives or the optimization of accountability; coal mining enterprises should focus on strengthening the construction of safety culture and safety supervision to reduce the vulnerability of the whole network. On this basis, efforts should be made to improve the skills of gas inspectors and pay attention to their physiological status, while focusing on protecting the physical environment of the system and the good operation of safety facilities.

Of course, this study is a research demonstration, and the application process of this method should be strictly followed in specific situations; only in this way can accuracy and scientific results be guaranteed.

## 4. Network Analysis Method for Human Errors and Its Application

As a part of safety science research, not only can accident causation models reflect the occurrence of certain types of accidents, but they also provide a scientific basis for the prevention and prediction of accidents, and the improvement of safety management [40]. In the past, there have been some quantitative analysis methods of accident causation modes based on accident cases (e.g., manuscript [27]), which reflect the rules of accident occurrence and find important causation factors, thus providing evidence for the targeted prevention of accidents. Evidence-based safety management has also become the representative of the new safety management method in the information age [41]. Therefore, based on the perspective of network analysis, this study proposes a new quantitative analysis method of analyzing human errors that occur to promote the targeted prevention of human errors.

### 4.1. Proposal of the Method

**Figure 9:**
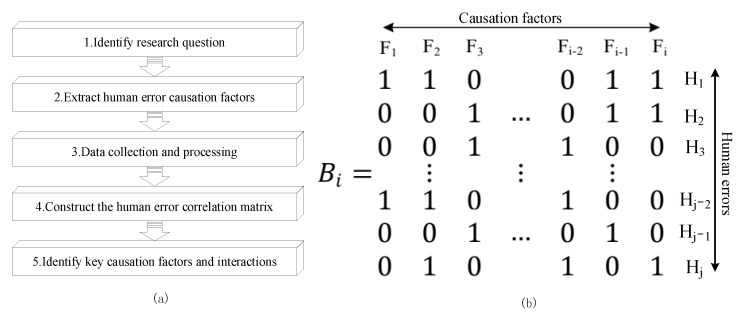
Human error causation data set and the overall process of this method (wherein, (**a**) is the whole process of network analysis of human error; (**b**) is the matrix formed by human error causation data).

This method collects and analyzes the existing accident cases on the basis of extracting the factors causing human error, and then makes quantitative analysis through a social network analysis (SNA). Among them, the SNA is a sociological research method to study actors and their relationships [39], which has also been used to analyze the influence degree of each structure in the social-physical system on the network, as well as accident causation factors and their relationships [42]. Therefore, this study uses this tool to conduct HFA by building a human error causation network and identifying key causative factors and interactions. The application of this method includes the following five steps: identification of research questions, extraction of human factors, data collection and processing, construction of a human error correlation matrix, and the identification of key causal factors and interactions. The overall process of this method is shown in Figure 9a. Based on the above process, the relevant elaboration is as follows:

(1) Identify research questions. Determining the research problem is the background application of this method, which should be refined into specific accidents within the region, specific industries, and specific systems. The identification of research questions is the basis for the identification of human error causes and data collection.
(2) Extract human error causation factors. The application of the method in Section 3 systematically summarized the causation factors of human error. On this basis, correcting the existing human error causation factors according to the specific situation needs to be applied.
(3) Collect and process data. The collection of accident cases is matched to the research question, and the scope or boundary usually involves the region, time, type of accident, severity of accident, etc. Among them, different regions may have different characteristics. For example, different countries and provinces have different safety systems and policies, which may lead to different characteristics of human mistakes; the accident case in the time period should have a positive guidance to the reality, as different accident severities may have different corresponding characteristics of human errors. For processing data, when analyzing a large number of accident cases, the process is usually carried out by more than one person. To improve the validity of the analysis results, all participants should be professionals with the sufficient knowledge structure. In addition, professionals involved in accident case analysis should be trained in consistency until “consistency ≥ 95%”. Additionally, the final results need to be reviewed and approved by relevant professors before subsequent analysis.
(4) Construct the human error correlation matrix. The purpose of this section is to study the co-occurrence of human error causation factors. To carry out targeted research, the construction of the human error correlation matrix is divided into safety managers and operators to obtain the corresponding data set of human error causation (if the influence factor exists, the value is “1”; otherwise, the value is “0”), as shown in Figure 9b. On this basis, relevant software (e.g., UNICET 6, NetMiner, MultiNet, etc.) is used to transform the data set of human error causation, and finally, the human error causation association matrix is obtained.
(5) Identify key causative factors and interactions. For the whole network, the density, average shortest path, and clustering coefficient of the human error causation association network explain the closeness, propagation, and cohesion of causation factors in the network [39]. For a single node, the degree centrality measures the direct influence of the causation factor on other factors, whereas the betweenness centrality measures the transmission control force of causative factors on the human error network [39]. Specifically, the greater the betweenness centrality, the more obvious the “bridge” effect of causation/association is.

### 4.2. Application of the Method

To verify the practicability and scientificity of this method in practice, this paper collected all the major and majorly significant coal mine gas explosion cases in China from 2010 to 2022 as examples to apply this method. Among them, the classification of accidents is based on three indicators, including the number of deaths (D), the number of serious injuries (I), and the economic loss (E), and any indicator within the corresponding range was judged. The determination scope in China of a major accident was determined by [43]: 10 ≤ D < 30; 50 ≤ I < 100; and 5 × 10^7^ RMB ≤ E < 1 × 10^8^ RMB. The determination scope in China of a particularly serious accident was also determined by [43]: D ≥ 30; I ≥ 100; and E ≥ 1 × 10^8^ RMB.

#### 4.2.1. Data Collection

This study was developed based on the official websites of relevant Chinese governments, including the Ministry of Emergency Management, PRC (https://www.mem.gov.cn, accessed on 1 March 2022) and the National Mine Safety Administration (https://www.chinamine-safety.gov.cn/, accessed on 2 March 2022). Based on the criteria in Table 3, a total of 39 major and particularly serious coal mine gas explosion accidents were collected in this paper, including a total of 41 management behavior errors and 52 operator behavior errors. Among them, the operators were mainly involved as blasting workers, ventilating workers, tunneling workers, and gas inspectors. Different individuals face different jobs and tasks; therefore, individuals need different qualifications and abilities, and different matters, environments, and management. Therefore, different types of workers were targeted and studied.

#### 4.2.2. Results

(1) A holistic analysis of human factor networks

Based on the association structure matrix, UCINET 6 was used to calculate the density, average shortest path, and clustering coefficient of the human factor network of different individuals. UCINET is by far the most commonly used comprehensive social network analysis tool, as it can perform an all-round analysis on the social network of individuals, structures, and processes, which completely matches the research purpose of this method. Among the factors [39], ➀ density refers to the ratio between the actual number of associations in the network and the maximum number in theory, which measures the tightness of the association between the causes of accidents in the network. The higher the value is, the closer the correlation between error causation is; ➁ the shortest path refers to the shortest distance traveled from one cause of an accident to another along the network. The average shortest path is the average of the shortest paths caused by accidents, which measures the propagation of accident causes in the network. The smaller the mean shortest path is, the more transmissible the error causation is; ➂ the clustering coefficient is measured based on the accessibility of the network. The higher the clustering coefficient, the stronger the cohesion of the network. The results are shown in Table 3.

The results show that: ➀ The density of the human factors network of all types of individuals is low, which indicates that the relationship between human factors in this network is low, and the influence path presented is not yet complex. In contrast, the human factor network density of tunneling workers and managers is higher. ➁ The average shortest path of the human factor network is lower for blasting workers and gas inspectors, but higher for tunneling workers and managers. Therefore, the latter is more transmissible. ➂ The clustering coefficient of the human factor network for ventilating workers and gas inspectors is higher, which indicates that the aggregation degree of the human factor is higher.

(2) Human factor network analysis of different individuals

**Figure 10:**
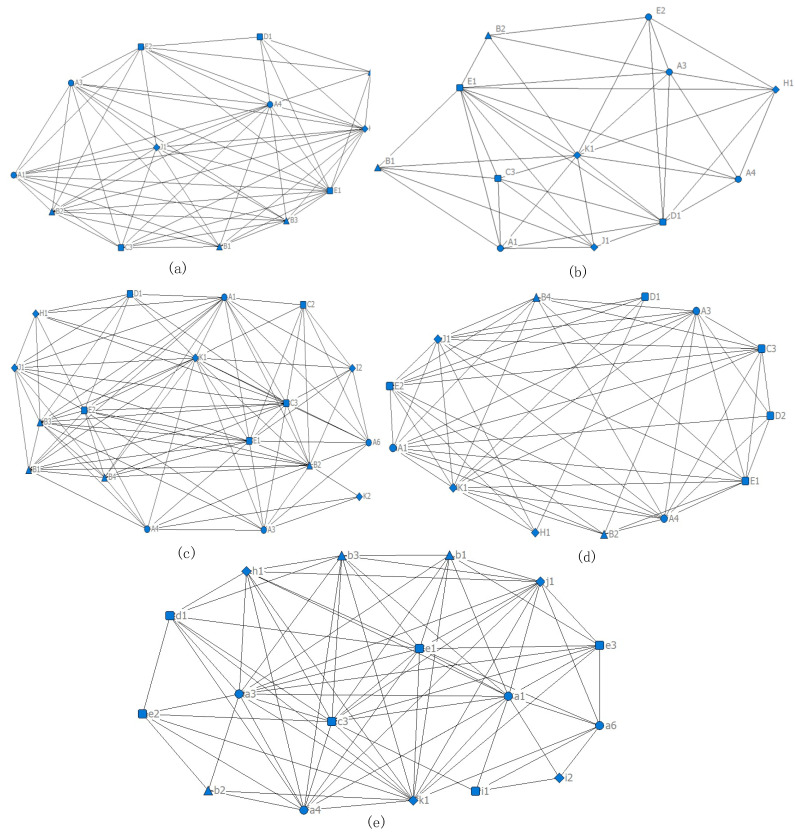
Human factor network analysis results of different individuals ((**a**–**e**) are the human error causation networks of blasting workers, ventilating workers, tunneling workers, gas inspectors, and managers respectively).

UCINET 6 software was also used to calculate the degree centrality, betweenness centrality, and edge betweenness centrality of five kinds of individual human error causation. On this basis, the top four/five causes/associations in each index were selected. By analyzing the values of these indicators, the key human error causations and correlations under each individual were identified, and then the human error mechanisms of different individuals were analyzed. The results are shown in Table 4. Human factor networks for different individuals are drawn (Figure 10a–e). These networks contain four types of nodes, the as same as the network nodes in Figure 8.

➀ For blasting workers, the main causes and paths of human error include: inadequate supervision at the government level (K1), insufficient enterprise supervision (E1), insufficient safety education and training (B2), and a not strict enough implementation of the access policy for blasting workers (E2) at the enterprise level, as well as skill defects at the individual level (A4). Among them, K1, A4, and E1 also have high centrality, indicating that these causative factors have a direct impact on other causative factors and play an important role in controlling the transmission of the human error causative network (Figure 10a).
➁ For ventilating workers, insufficient supervision at the government level (K1), insufficient supervision (E1), insufficient investment in ventilation equipment (H1), lack of safety education (B1) at the enterprise level, and deficiency of knowledge at the individual level (A3) are the main factors and pathways leading to human error. Under the premise of weak government supervision, this leads to further lax internal safety and ventilation work in enterprises, mainly involving the delay of safety information feedback, the lack of knowledge and skills training, the lack of enterprise supervision, and the reduction of ventilation access standards (Figure 10b).
➂ For tunneling workers, the lack of supervision at the government level (K1), the lack of supervision (E1), the lack of a safety climate at the enterprise level (C3), unsafe psychology at the individual level (A1) and the defect of knowledge (A3) are the main causes and paths leading to human error. At the same time, an important intermediary link also includes SIS at the enterprise level (B2). In the case of government regulation violation, the failure of SIS and the lack of safety knowledge and skills is more serious (Figure 10c).
➃ For gas inspectors, the lack of supervision at the government level (K1), the lack of strict implementation of access standards at the enterprise level (E2), as well as the unsafe psychology (A1), the defect of knowledge (A3), and the defect of skills (A4) at the individual level are the main factors and paths leading to human error. In addition, the lack of corporate constraints on the cultural environment is the key cause of the unsafe psychology of gas inspectors. At the same time, when government supervision is insufficient or the enterprise safety climate is insufficient, it has a negative impact on the enterprise’s investment in gas measurement equipment (Figure 10d).
➄ For managers, the lack of supervision at the government level (K1), the lack of safety supervision (E1) and the lack of a cultural environment (C3) at the enterprise level, as well as unsafe psychology at the individual level (A1), the lack of knowledge (A3), and the lack of skills (A4) are the main factors and pathways leading to human error. In addition, the failure of managers to protect the cultural environment further stimulates the generation of unsafe psychology; the quality of the cultural environment is also directly related to whether managers destroy it. At the same time, in the absence of government supervision, the bad habits of managers and the defects of SIS of enterprises becomes more serious (Figure 10e).

From the above results, it can be seen that the causes and paths of the above human factor network are presented as the joint influence of macro-, meso-, and micro-causes. The difference is that the influencing factors and paths of different individuals are different. In addition, there are some nodes that have not yet appeared in the case selected in this paper. We believe that this does not affect the scientific research of this paper, and the characteristics of factors affecting human error in different systems and accidents are always different. As a result, the above results provide an important basis for the prevention of specific human errors.

## 5. Discussion

HFA is an important means to prevent human factor error effectively. The HFA based on a complex network proposed in this paper formally introduced network analysis tools into HFA. This is a response to the complexity of the current system. This study constructed the human error causation mode corresponding to the research situation in this paper, and on this basis, proposed the system safety method of HFA and the network analysis method of human error.

(1) The human error causation mode proposed in this paper is an integrated mode, involving signal-noise dependence, information dependence, and constraint dependence, and comprehensively summarizes all the factors that may affect safety behavior. These factors originate from humans, matters, environments, management, and their interactions. The human error causation models proposed in the past (such as the HFACS [13] and SICHFA [2]) have their focus (the former focuses on humans and management, whereas the latter focuses on information and cognition), but they are not applicable to the current complex network system. The human error causation model is comprehensive and universal, but it also has limitations: the mode constructed in this paper is a preliminary research result, which needs to be further improved.
(2) The system safety method of HFA also has its own characteristics, which cannot only clarify the direct coupling effect of various elements in the system, but also can promote the visualization of human factors through the network graph so that all subjects can effectively participate in the prevention of human error. On the one hand, vulnerable points can be identified according to the complex network graph; on the other hand, the network analysis tool can be used to evaluate the key nodes in the system and provide an important basis for prevention work. Therefore, this method combines both qualitative and quantitative attributes, and can promote the initiative, systematic, and dynamic prevention of human error. In the future, an intelligent platform can be introduced to promote the intelligentization of human error prevention. However, the difficulty (operability, workload, etc.) of applying this method will be a major obstacle in practice.
(3) For the network analysis method of human error, compared with the previous purely descriptive literature research, this method not only can identify the nodes with the highest frequency of the effects in certain people due to errors, but also can spread the risk and play an important intermediary role in the network of nodes and edges, which can be effective for recognition. Therefore, this method will be more effective in preventing human error. Its limitation lies in that the method does not fully play the role of network tools, which is needed to solve more problems and further promote the prevention of human error. Subsequently, network tools can be used to mine more information about human error, such as: How does risk spread in the network? Are there other values that can characterize the key features of human error? How does the system network become an optimal network?

By comparing the application of the two methods, we found that there is partial overlap in the application results, which reflects the difference between theory and practice, and these two methods are complementary to some extent. Therefore, the combination of the two methods will make the prevention of human error more effective.

## 6. Conclusions

Aiming at the complexity of the system and coupling of elements, a new HFA method based on a complex network was proposed in this paper. This method was based on the lack of or incomplete studies on the indirect effects of human error. Therefore, this paper first proposed am HFA network which contains three influencing factors (signal noise, information, and constraint) of human error. On this basis, the system safety method of HFA and the network analysis method of human error were proposed. These two methods are complementary in function, as the former is a safety analysis for the whole system, which can find all possible risk points, vulnerable points, and risk propagation paths, whereas the latter is a targeted analysis based on existing data. At the same time, this paper also applied the above methods to the analysis of human factors in gas explosion accidents, and the application results can provide an important basis for the prevention of related human errors.

Although past research has covered many of the contents of human factor analysis, it also has some practical value. However, the current situation of human error is still not optimistic enough. In a complex safety system, all system components are nodes of a complex network. The authors believe that it is necessary to combine the correlation between each node to study the human error, which is the real state of the human safety behavior affected by the system. Therefore, the authors call on more scholars to join the camp of HFA research from the perspective of a complex network.

## Figures and Tables

**Table 1:** Causation of human error from the perspective of complex network.

Causation Link	Causative Analysis of Operator’s Behavior Error	Causative Analysis of Safety Manager’s Behavior Error
Human	A1: bad psychology; A2: adverse physiology; A3: defects of knowledge; A4: defects of skill; A5: physical/mental defects; A6: bad habit; A7: inadequate personal preparation.	a1: bad psychology; a2: adverse physiology; a3: defects of knowledge; a4: defects of skill; a5: physical/mental defects; a6: bad habit; a7: inadequate personal preparation.
Human ⟷ Human	B1: failure of SIF; B2: failure of SIS; B3: failure of SIE; B4: weak supervision.	b1: failure of SIF; b2: failure of SIS; b3: failure of SIE.
Environment → Human	C1: unsafe physical environment; C2: unsafe technical environment; C3: unsafe cultural environment.	c1: unsafe physical environment; c2: unsafe technical environment; c3: unsafe cultural environment.
Matters → Human	D1: lack of safety matters; D2: failure of safety matters.	d1: lack of safety matters; d2: failure of safety matters.
Enterprise → Human	E1: weak supervision; E2: unreasonable access and arrangement.	e1: weak supervision; e2: improper plan; e3: failure to correct problems in time.
Environment ⟷ Matters	F1: unsafe physical environment; F2: lack of safety matters; F3: failure of safety matters.	f1: unsafe physical environment; f2: lack of safety matters; f3: failure of safety matters.
Human → Matters	G1: the action of damaging safety matters; G2: poor protection of safety matters.	g1: the action of damaging safety matters; g2: poor protection of safety matters.
Enterprise → Matters	H1: under-investment in safety; H2: safety resources are improperly allocated; H3: inapplicable safety matters.	h1: under-investment in safety; h2: safety resources are improperly allocated; h3: inapplicable safety matters.
Human → Environment	I1: the action of damaging safety environment; I2: poor protection of safety environment.	i1: the action of damaging safety environment; i2: poor protection of safety environment.
Enterprise → Environment	J1: insufficient constraints on the safety environment.	j1: insufficient constraints on the safety environment.
Government → Enterprise	K1: weak supervision; K2: regulatory violations.	k1: weak supervision; k2: regulatory violations.
Matter → Matter	L1: cascading effect between matters.	l1: cascading effect between things.

**Table 2:** Causation of gas inspectors’ behavioral errors from the perspective of a complex network.

Node	Causation Factor	Node	Causation Factor
A1	Fluke psychology, experience psychology, neglect psychology, lack of motivation, and so on.	E1	Coal mine enterprises have insufficient supervision of dispatching personnel.
A2	Acute physical conditions, such as physical discomfort.	E2	Mining companies employ unqualified gas inspectors.
A3	Lack of professional knowledge and system cognition about disaster prevention and mitigation of gas explosion.	F1	High temperature, humidity, and dust cause device failure.
A4	Usage error of test instrument; screening error of potential hazard; system execution capacity is insufficient.	F2	Lack of equipment to regulate the working environment, such as air conditioning.
A5	Permanent physical or mental disability, such as organ defects, intelligence, etc.	F3	The equipment regulating the working environment is damaged or aging.
A6	Spontaneous unsafe behavior, such as poor testing habits.	G1	Human behavior causes damage to air conditioners, gas ventilation, and monitoring equipment.
A7	Inadequate mental and physical preparation, such as rest, alcohol restriction, etc.	G2	Insufficient maintenance and protection of safety facilities by managers.
B1	Other personnel did not timely inform the behavior defects of human factors in gas monitoring.	H1	Enterprises have not invested enough in gas drainage, monitoring equipment, and other equipment to regulate the environment, resulting in insufficient quantity and poor efficiency.
B2	Safety education and training are inadequate.	H2	Equipment is unevenly distributed among coal mines.
B3	The communication of potential hazard identification, equipment status, and other fuzzy information is delayed or invalid.	H3	Safety facilities are not fully applicable, such as fire extinguishers.
B4	Lack of adequate supervision and restraint on their behavior.	I1	Individual neglect of safety affects the safety climate.
C1	Physical effects of temperature, noise, etc.	I2	Insufficient maintenance and protection of safety environment by managers.
C2	The design and display of instruments, equipment and facilities are not conducive to the smooth development of behaviors.	J1	The overall organizational climate of culture, policy, and strategic direction is lacking.
C3	Lack of safety climate.	K1	Inappropriate oversight and supervision of personnel and resources.
D1	Gas drainage, and monitoring and other equipment is insufficient.	K2	Management willfully flouted procedures, regulations, and policies.
D2	Gas drainage and monitoring equipment are damaged or outdated.	L1	Damage to the physical structure leads to the failure of other physical structure functions, such as a power outage that leads to the failure of equipment throughout the mine.

**Table 3:** Overall analysis results of the HFA network.

Individuals	Density	Average Shortest Path	Clustering Coefficient
Blasting workers	0.1411	1.333	0.867
Ventilating workers	0.0907	1.394	0.956
Tunneling workers	0.1885	1.451	0.819
Gas inspectors	0.1179	1.346	0.973
Managers	0.1623	1.456	0.834

**Table 4:** Node analysis results of HFA network.

Individuals	Degree Centrality					Betweenness Centrality					Edge Betweenness Centrality			
Blasting workers	K1	A4	E1	B2	E2	E1	K1	A4	J1	A1	E1-D1 (5)	E1-A1 (4.833)	E1-A3 (4.833)	B4-A4 (3)
Ventilating workers	K1	E1	A3	D1	H1	K1	E1	D1	A3	E2	K1-B1 (4.2)	E1-B2 (4.033)	K1-E2 (3.95)	K1-B2 (3.833)
Tunneling workers	K1	E1	C3	A1	A3	B2	K1	C3	A1	E1	K2-B2 (8.588)	K2-A3 (4.263)	K2-A4 (4.149)	J1-D1 (3.533)
Gas inspectors	K1	A3	E2	A4	A1	K1	E2	J1	A3	A1	K1-D1 (4.024)	K1-H1 (4.024)	J1-DI (3.524)	J1-HI (3.524)
Managers	k1	a1	a3	c3	e1	a1	k1	c3	a3	a4	i2-a1 (10.617)	i1-c3 (8.267)	k1-a6 (6.242)	k1-b2 (5.875)

## Data Availability

The data presented in this study are available on request from the corresponding author.
